# Ultrasound for Appendicitis: Performance and Integration with Clinical Parameters

**DOI:** 10.1155/2016/5697692

**Published:** 2016-12-01

**Authors:** Fanny Löfvenberg, Martin Salö

**Affiliations:** Department of Clinical Sciences, Pediatrics, and Department of Pediatric Surgery, Lund University, Skåne University Hospital, Lund, Sweden

## Abstract

*Objective*. To evaluate the performance of ultrasound in pediatric appendicitis and the integration of US with the pediatric appendicitis score (PAS) and C-reactive protein (CRP).* Method*. An institution-based, retrospective study of children who underwent abdominal US for suspected appendicitis between 2012 and 2015 at a tertiary pediatric surgery center. US results were dichotomized, with a nonvisualized appendix considered as a negative examination.* Results*. In total, 438 children were included (mean 8.5 years, 54% boys), with an appendicitis rate of 29%. The sensitivity, specificity, positive predictive value (PPV), and negative predictive value (NPV) for US were 82%, 97%, 92%, and 93%, respectively, without significant age or gender differences. Pediatric radiologists had significantly higher sensitivity compared to general radiologists, 88% and 71%, respectively (*p* < 0.01), but no differences were seen for specificity, PPV, and NPV. The sensitivity, NPV, and negative likelihood ratio for the combination of negative US, PAS < 5, and CRP < 5 mg/L were 98%, 98%, and 0.05 (95% CI 0.03–0.15).* Conclusion*. US may be a useful tool for evaluating children with suspected appendicitis, regardless of age or gender, and should be the first choice of imaging modalities. Combining US with PAS and CRP may reduce several unnecessary admissions for in-hospital observation.

## 1. Introduction

Despite the high incidence of appendicitis during childhood, the diagnosis remains difficult with risk of diagnostic delay and perforation [1], as well as negative appendectomies [2]. Despite a higher negative appendectomy rate in girls [2–5], imaging is frequently more used in this group [6–8]. Perforated appendicitis and negative appendectomy have a reported frequency of 17–35% [4, 9–11] and 1–12%, respectively [4, 7, 8, 10]. When introducing preoperative imaging for suspected appendicitis in children, there were hopes for a reduction of perforations and negative appendectomies; nevertheless the literature is showing inconsistent results [5, 7].

The reported sensitivity for US varies in the literature (67–100%) [10–14] and is lower than computed tomography (CT) [10, 11]. However, the awareness of the lifetime risk for radiation induced malignancy in children has decreased the use [15]. Despite the variety in the sensitivity, the reported specificity of US is 95–98% [10–14], which is almost equal to the specificity of CT [11]. Taken together, US is most often the first line imaging modality for suspected appendicitis in children [10, 16]. Factors that could affect the variation in sensitivity of US is a field of interest. One study showed a lower sensitivity in girls [6]. Another factor is the operator dependency of US. An equivocal result, when appendix is partly or not visualized, is not uncommon while using US [8, 12, 13, 16, 17]. The operator-dependent nature of US can possibly explain some of the discrepancy in sensitivity between US and CT [18]. A higher frequency of identifying appendix in children has been shown to be related to the hospitals' experience from using US regularly [19], and if the US examiner has pediatric expertise [12]. In addition, a higher identification rate of appendix would most likely increase the sensitivity and specificity of US for suspected appendicitis [19].

In order to improve the diagnostic accuracy for US, identifying clinical predictors for US could be of help, especially for an equivocal US. Hypothetical clinical predictors for US could be age, sex, lab measurements, or a clinical prediction score, such as Pediatric appendicitis score (PAS) or Alvarado Score. In addition, only a few studies have considered the possibility of combining US with a clinical prediction score (Table 1) [20–23]. Furthermore, studies have evaluated the combination of routine blood tests such as C-reactive protein (CRP) and white blood cell (WBC) count, with US (Table 1) [24, 25].

The aim of this study was to evaluate how gender, age, and operator experience affect the diagnostic performance of US in children with suspected appendicitis. Secondary aim was to examine if integration of US with PAS and CRP could be used to exclude the diagnosis of appendicitis with high safety in a substantial number of patients.

## 2. Patients and Methods

This study was approved by the Regional Ethical Review Board (registration number 2010/349).

### 2.1. Settings and Children

The cohort consisted of patients <15 years of age who underwent abdominal ultrasound for suspicion of appendicitis from 2012 to 2015 at a university hospital. The hospital covers an area of 340.000 inhabitants with care for children with acute abdominal pain. Children who present with acute abdominal pain in the pediatric ED are seen by a pediatrician or by a pediatric surgeon, depending on the initial triage or, if admitted from the primary care, on what specialty the child is admitted to.

### 2.2. Study Design

A retrospective study was conducted using a database of all children who had an abdominal ultrasound between 2012 and 2015. The inclusion criteria were <15 years of age, seeking the pediatric ED with acute abdomen, and abdominal US for suspected appendicitis. Suspicion of appendicitis had to be documented on the imaging request of the patient, for inclusion in the study. Patients already hospitalized and with acute abdomen presenting during treatment for other conditions were excluded. The medical records of the included patients were studied and the following parameters registered: age, gender, duration of symptoms, patient history, findings from the physical examination, results from routine blood tests (WBC, neutrophils, and CRP), experience of referring doctor, experience of radiologist, result from the US examination, and patients' final diagnoses. Based on the patient history, abdominal examination, and laboratory tests, PAS was calculated for each patient. The normal routine in our department is that the diagnosis and degree of appendicitis are based on the surgeon's description and in equivocal cases on histopathology.

### 2.3. Definitions and Classifications

Duration of symptoms was calculated from onset of symptoms to US examination. Symptoms, findings from the abdominal examination, results from routine blood tests, and PAS were registered close in time to the US examination. Hence, some children underwent several abdominal examinations and lab measurements. PAS is a 10-point clinical prediction score, developed to measure the probability of appendicitis in children [26]. The eight components of PAS are anorexia, nausea/vomiting, pain migration, fever, leukocytosis, left shift on WBC differential, RLQ tenderness, and cough/percussion/hopping tenderness in the RLQ [26]. Each of these components scores one point, except the two parameters describing tenderness in the RLQ which give two points each. The referring physician admitting the child for an ultrasound was categorized as pediatric surgeon or pediatrician and as resident or specialist. The radiologist performing the ultrasound was classified as pediatric radiologist or general radiologist; no technologists were involved. US results were categorized as positive or negative, since the results from a clear dichotomized “answer” would be of most use for the physician. A positive US was defined as visualization of an appendix with signs of appendicitis. Terms like “suggestive appendicitis” or “suspicion of appendicitis” were categorized as positive. During the study period, the general ultrasound criteria for appendicitis were thickness >6 mm, together with other possible signs such as hyperemia, free fluid, signs of obstruction, noncompressible appendix, and pain when applying pressure with the transducer. A negative US was defined as visualization of a normal appendix or as a nonvisualized appendix without secondary signs of appendicitis. The degree of appendicitis was classified as phlegmonous, gangrenous, or perforated appendicitis or appendiceal abscess.

### 2.4. Statistical Analysis

Statistical analyses were performed using SPSS Statistics, version 22. Fisher's two-tailed exact test was used for dichotomous variables when comparing two groups. When comparing sensitivity, specificity, and predictive values between several groups, chi-square test with post hoc test was used. When comparing differences between children with appendicitis and a positive and negative ultrasound, respectively, and differences between patients with a visualized appendix or not, logistic regression was used. The CRP values were logarithmized in the logistic regression since they did not have a normal distribution. Receiver operating characteristics (ROC) curve was performed for US, PAS, and CRP. From the ROC curve, the best cut-offs for PAS and CRP were retrieved for use in the integration with US and PAS, with the purpose of ruling out appendicitis in the highest numbers of patients possible. Patients lacking elements of PAS and/or laboratory values were excluded in the evaluation of the scores. A *p* value < 0.05 was considered statistically significant.

## 3. Results

### 3.1. Description of Cohort

A total of 6454 patients underwent abdominal ultrasound from 2012 through 2015, of which 438 patients (8.5 ± 3.4 (mean, SD) years, 54% boys) matched the inclusion criteria. Of these, 125 (29%) had a final diagnosis of appendicitis, and 313 (71%) children other final diagnoses. The degree of appendicitis was 74 (59%) phlegmonous, 20 (16%) gangrenous, 21 (17%) perforated, and 10 (8%) with appendiceal abscess which is shown as follows.

The following is overview of final diagnoses in 438 children who underwent ultrasound for suspicion of appendicitis.

*Nonappendicitis group (N = 313)*

Unspecified abdominal pain (159)
Mesenterial lymphadenitis (47)
Constipation (22)
Gastroenteritis (11)
Pyelonephritis (11)
Pneumonia (9)
Terminal ileitis (7)
Undiagnosed infection (5)
Ruptured ovarian cyst (5)
Ovulation (5)
Urinary tract infection (5)
Tonsillitis (4)
Viral infection (4)
Sepsis (3)
Infected urachus (2)
Hydronephrosis (2)
Cholecystitis (1)
Pancreatitis (1)
Intussuception (1)
Meckels diverticulum (1)
Intra-abdominal vascular malformation (1)

*Appendicitis (N = 125)*

Phlegmonous (74)
Gangrenous (20)
Perforated (21)
Abscess (10)

Appendectomy was performed in 118 children with appendicitis, while seven patients with an appendiceal abscess were conservatively treated. The negative appendectomy rate was 10% (12/118).

Of the 237 boys, 74 (31%) had appendicitis, and of the 201 girls, 51 (25%) had appendicitis. The mean duration for onset of symptoms to US examination was 44 ± 39 and 55 ± 44 hours for the appendicitis group and the nonappendicitis group, respectively. PAS and CRP were 6.3 ± 1.9 and 25 (5–431) mg/L for children with appendicitis and 3.8 ± 1.9 and 5 (5–382) mg/L in the nonappendicitis group (Table 2). ROC curve analysis showed an AUC for PAS of 0.80 (95% CI 0.75–0.86) at a cut-off of ≥6 with no other cut-offs having better AUC and for CRP 0.64 (95% CI 0.58–0.71) with ideal value of 15 mg/L.

### 3.2. Evaluation of Ultrasound Performance

**Figure 1:**
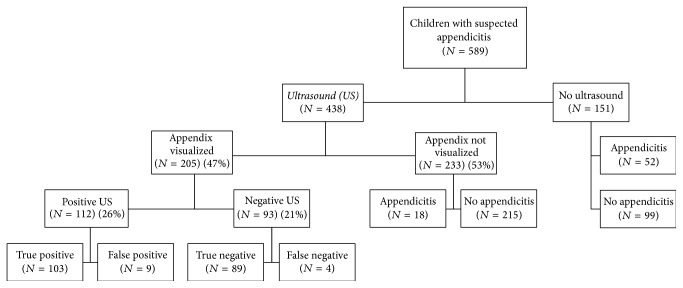
Flowchart of ultrasound results in 438 children with suspected appendicitis.

Appendix was visualized in 205 (47%) of the 438 enrolled patients. Of those with a visualized appendix and negative US, 4% had appendicitis. In the 233 cases when appendix was not visualized and no secondary signs of appendicitis were seen, 8% had appendicitis (Figure 1). The visualization rate of appendix, for pediatric and general radiologists, was 49% and 44%, respectively (*p* = 0.33). When evaluating odds ratios (OR) for visualization of the appendix, appendicitis had an OR of 11.8 (95% CI 5.8–24.2) (*p* < 0.01), and CRP an OR of 1.9 (95% CI 1.2–3.0) (*p* < 0.01). No significant differences were seen for age, gender, duration of symptoms, PAS, or experience of the radiologist.

Overall, the sensitivity, specificity, PPV, and NPV for ultrasound were 82%, 97%, 92%, and 93%, respectively, and the AUC was 0.88 (95% CI 0.83–0.93). The positive and negative likelihood ratio (LR) was 28 (95% CI 15–55) and 0.18 (95% CI 0.12–0.26), respectively. There were no significant differences between genders or age groups. No differences in OR could be seen when evaluating age, sex, duration of symptoms, experience of the radiologist, PAS, or CRP, in children with appendicitis with comparison of patients with positive versus negative ultrasound. Pediatric radiologists had significantly higher sensitivity in US performance compared to general radiologists, 88% and 71%, respectively, (*p* < 0.01), while no significant differences were seen when comparing specificity, PPV, or NPV (Table 3).

Referring pediatric surgeons had a significantly higher rate of positive US than pediatricians, 36% and 19%, respectively (*p* = 0.03). No difference was seen when comparing referring residents and specialists, 30% and 25%, respectively (*p* = 0.21).

### 3.3. Ultrasound, PAS, and CRP

No patient with a PAS of 0–3 had appendicitis. Among patients with PAS 4–6 and a negative US, 7% had appendicitis, and among patients with a negative US and CRP < 15 mg/L, 5% had appendicitis. Two scores, with the purpose of ruling out appendicitis, were created, with integration of US with PAS and/or CRP. Patients lacking data for PAS (*N* = 36) and CRP (*N* = 15) were excluded from the evaluation of the scores. The sensitivity and NPV for negative US and CRP < 15 mg/L was 91% and 95%, respectively, with a negative likelihood ratio (LR) of 0.14 (0.08–0.26). The sensitivity and NPV for negative US, PAS ≤ 5, and CRP < 5 mg/L were both 98%, and the negative LR was 0.05 (95% CI 0.03–0.15) (Table 4).

## 4. Discussion

US seems to be a useful tool for evaluating children, regardless of age or gender, with suspected appendicitis. When US is integrated with PAS and/or CRP, a high NPV can be reached for a substantial part of the patients.

### 4.1. Diagnostic Performance

US for suspected appendicitis had a sensitivity of 82% for the entire cohort, which is similar to other studies [7, 10], though lower [12, 19, 22], and higher [11, 13, 14, 16], values have been reported. The difference in sensitivity between studies can have several explanations. The classification of US results is not consistent among studies; a nonvisualized appendix can be categorized as equivocal or negative. The binary categorization in the present study, with a nonvisualized appendix without secondary signs of appendicitis classified as a negative examination, is in conjunction with some of the previous studies [12, 21, 24]. Conversely, Schuh et al. [17], classified a partly or nonvisualized appendix without secondary signs, as equivocal. Further, Mittal et al. [19] described that institutions using US regularly had a higher sensitivity, and the sensitivity increased with increased visualization of appendix. Trout et al. [12] explored the different classification options and found a sensitivity of 67%, when including a nonvisualized appendix as a negative examination, and a sensitivity of 99%, when only including cases with a visualized appendix. The visualization rate of appendix varies within a large range of 24–73% [12, 19]. Our rate of 47% is close to a 48% visualization rate found in a large multicenter cohort [19]. In conclusion, the sensitivity in the present study might have been higher with a different classification of the US result, but when considering a physician's perspective, a binary classification is desirable for the practical clinical management of patients.

Gender differences in US for appendicitis have been reported, with lower sensitivity for girls, with the explanation that US in girls primarily is used to exclude gynecologic diseases [6]. However, no difference was found between boys and girls in the present study, and only five of the girls were diagnosed with a gynecologic diagnosis. The PPV and NPV, 92% and 93%, respectively, are in the higher range compared to other studies [10, 13, 14, 17]. As known, when interpreting predictive values, it is important to consider the prevalence of the disease. In this present study, 29% had appendicitis, which is similar to some studies [11], but higher than others [12, 13]. Consequently, studies with a lower prevalence of appendicitis had a low PPV (75–82%) and a high NPV (93–98%) [12, 13]. Further, the specificity of 98% is in line with some studies [12, 19] and somewhat higher than others [11, 21].

### 4.2. Operator Experience

Pediatric radiologists were found to have statistically higher sensitivity than general radiologists. Interestingly, the visualization rate of appendix did not differ significantly between the two groups. A higher performance and visualization rate of appendix, with pediatric expertise, have been described previously [12]. However, the visualization rate of appendix was quite low (24%), and the US was performed by sonographers, with interpretation of the images by radiologists [12]. On the other hand, no differences were seen between the two groups in specificity, and more importantly, PPV and NPV. Hence, despite the difference in sensitivity, the present study does not support a major difference between general and pediatric radiologists.

Regarding the significantly higher rate of positive US for pediatric surgeons seen in this study, one could speculate that pediatric surgeons use US in greater extent to confirm a high suspicion of appendicitis, whereas pediatricians use US to rule out appendicitis. Another possibility is that pediatric surgeons are better at triaging patients to ultrasound, hence admitting children with a higher pretest probability.

### 4.3. Integration of US with Clinical Parameters

To our knowledge, there are six previous studies evaluating integration of ultrasound with clinical parameters for diagnosis of appendicitis in children [20–25]. No study has evaluated CRP integrated with US. Zouari et al. did not find it helpful when integrating CRP with the Alvarado score [24]. In the present study, integrating a negative US and CRP < 15 mg/L improved sensitivity (82% to 92%) and slightly increased the NPV (93% to 95%). Another study found that incorporation of WBC count and PMN% could substantially improve the predictive values of US [25]. We did not specifically look at these laboratory values but both WBC count and neutrophils are a part of PAS.

Two studies have evaluated integration of PAS and US [20, 21]. In our study, among children with PAS 0–3, none had appendicitis with a negative or positive US. Similar analysis for negative US was made by Bachur et al. [21], but with a false-positive rate of 27% (for US) in the PAS 0–3 group. Among patients with PAS 4–6 and a negative US, 7% had appendicitis, which also is similar to the study by Bachur et al. [21]. Another study found that a cut-off at PAS ≤ 5 could identify patients with an equivocal US that had low likelihood of appendicitis [20]. In the present study, false-negative US examinations increased with increasing PAS, also described by others [21]. Children with PAS 7–10 had a false-negative rate of 31% with a negative US, compared to a rate of 19% in the study by Bachur et al. [21].

From the present study and other studies evaluating integration of US with PAS [20, 21], or with Alvarado score [20, 22–24], and from recent guidelines on pediatric appendicitis [27, 28], it seems that patients should be categorized into three different groups based on the clinical prediction score: one group with low probability (0–3 points), one with intermediate probability (4–6 points), and one group with high probability of appendicitis (8–10 points). It seems that children in the low probability group could safely be sent home without US. Children with intermediate probability should undergo US and based on the result be sent home if the US is negative. If there is still a clinical suspicion of appendicitis, the children may be scheduled for a followup visit. Children in the high probability group may not benefit from an US since the rate of false-negative results seems to increase which may mislead the surgeon. Hence, in presence of a high clinical prediction, the physician has different options, where the two most accurate seem to be active observation or diagnostic laparoscopy according to the most recent published guidelines [27, 28].

In order to decrease the false-negative rate, we integrated US with both PAS and CRP. The score with following parameters, PAS ≤ 5, CRP < 5 mg/L, and a negative US, could almost rule out appendicitis. The sensitivity and NPV for this clinical pathway were both 98%, and the negative LR 0.05, compared to US alone which had a NPV of 93% and a negative LR of 0.18. Further, a NPV of 99% has been reported for a nonvisualized appendix using CT [29], and in the present study, negative US included the cases with a nonvisualized appendix. In conclusion, a NPV of 98%, with the suggestive clinical management, is close to and does not have the disadvantages of CT. One could argue that the difference between a NPV of 93% (US alone) and 98% is not significant. However, since suspicion of appendicitis is common, even a small improvement of the diagnosis can have a substantial impact. Further, the necessary clinical information and routine blood tests, to combine PAS and CRP with US, are often a part of the basic workup in children with suspected appendicitis and hence do not require any special resources. Therefore, this clinical score, combining PAS, CRP, and US, seems useful and easily applicable to safely rule out appendicitis for a substantial part of children seeking the pediatric ED with abdominal pain.

If further imaging is indicated after US, CT has become the second line imaging modality. However, due to the potential risk of malignancy [15, 30], serial US [31] and magnetic resonance imaging (MRI) [32, 33] have recently been suggested as alternatives to CT. A meta-analysis proposed MRI as an optional first line imaging modality for suspected appendicitis in children [32]. However, availability, cost, and the possible need for sedation in young children make the practical use of MRI called into question. Further, American College of Radiology recommend CT in negative or equivocal cases, although MRI is mentioned as a future alternative [30].

### 4.4. Limitations

In a retrospective study, missing data is not rare. Further, information bias for calculating some of the elements in PAS cannot be excluded. However, only one person was responsible for collecting data, minimizing the risk of interrater bias. Also, patients lacking data for PAS or blood tests would not have been seen in that extent if a prospective study would have been conducted. At last, one plausible limitation to the present study is the binary classification of US. The knowledge of varying US results between institutions and the fact that this is a single-center study limit the use of our results. However, our US results are in line with reported values from a large meta-analysis [11].

## 5. Conclusion

US may be a useful tool for evaluating children with suspected appendicitis, regardless of age or gender, and should be the first choice of imaging modalities. It seems that suspicion of appendicitis can be ruled out in a substantial number of patients when US is integrated with PAS and CRP.

## Figures and Tables

**Table 1:** Overview of studies evaluating integration of ultrasound with clinical parameters for pediatric appendicitis.

Study	Patients (*N*)	Integration with	Conclusion
Athans et al.	776	Alvarado PAS	If equivocal US examination was used, a low clinical score (≤5) may be used to identify patients with a low likelihood of appendicitis.

Bachur et al.	728	PAS	False-negative US increase with increasing PAS, and false-positive US occur more often with lower PAS. Discordance between US results and clinical assessment warrants serial examinations or further imaging.

Blitman et al.	522	Alvarado	Children with inconclusive US and low Alvarado score (<5) are extremely unlikely to have appendicitis.

Toprak et al.	122	Alvarado	In children with a nonvisualized appendix and without a high Alvarado score, appendicitis can be safely ruled out.

Zouari et al.	292	Alvarado CRP	Integration of Alvarado score and ultrasound improve the predictive values of diagnosing appendicitis.

Anandalwar et al.	845	WBC count PMN	Integration of US with WBC count and PMN% can substantially improve the predictive values of diagnosing appendicitis.


PAS: pediatric appendicitis score; CRP: C-reactive protein; US: ultrasound; WBC: white blood cell; PMN% = polymorphonuclear leukocyte differential.

**Table 2:** Demographics, duration of symptoms, and clinical data in patients with ultrasound for suspicion of appendicitis.

	Appendicitis (*N* = 125)	Not appendicitis (*N* = 313)
Age (years)	8.8 ± 3.5	8.4 ± 3.2
Gender (M/F)	74/51	163/150
Duration of symptoms (h)	46 ± 34	53 ± 41
PAS (0–10)	6.4 ± 1.6	3.8 ± 1.7
CRP (mg/L)	25 (5–431)^a^	5 (5–382)^b^

Values presented as mean ± SD (standard deviation) or median (min–max); PAS: pediatric appendicitis score; CRP: C-reactive protein; patients lacking PAS (*N* = 36) or CRP (*N* = 15) were not included; a: 24 patients with normal value; b: 164 patients with normal value.

**Table 3:** Diagnostic performance of ultrasound for appendicitis with regard to gender, age group, and experience of examiner.

	Diagnostic performance% (95% CI)				LR+/LR− (95% CI)
	Sensitivity	Specificity	PPV	NPV	
All patients	82 (75–89)	97 (94–99)	92 (85–96)	93 (90–96)	28 (15–55)/0.18 (0.12–0.26)
Boys	83 (72–91)	98 (94–100)	93 (84–99)	92 (87–96)	
Girls	80 (67–92)	97 (94–99)	91 (78–99)	93 (89–98)	

Age group (years)					
0–4	74 (45–92)	96 (84–100)	92 (66–100)	89 (75–96)	
5–9	86 (71–95)	100 (96–100)	100 (89–100)	95 (90–98)	
10–14	81 (67–91)	95 (90–99)	88 (74–96)	92 (87–95)	

Examiner					
Pediatric radiologist	88 (76–95)	98 (93–100)	94 (86–99)	95 (90–99)	
Radiologist	71 (56–84)	97 (94–99)	90 (74–98)	91 (86–95)	

PPV: positive predictive value; NPV: negative predictive value; CI: confidence interval; LR: likelihood ratio.

**Table 4:** Integration of ultrasound with pediatric appendicitis score (PAS) and C-reactive protein (CRP) in the diagnosis of pediatric appendicitis.

	US positive *N* (% appendicitis)	US negative *N* (% appendicitis)	Patients (*N*)
PAS			
0–3	7 (0%)	116 (0%)	123
4–6	59 (93%)	143 (7%)	202
7–10	40 (100%)	37 (31%)	77

CRP (mg/L)			
<15	36 (89%)	191 (5%)	227
≥15	74 (91%)	122 (8%)	196

PAS ≤ 5 + CRP < 5	9 (50%)	118 (3%)	127

Diagnostic performance% (95% CI)			

Negative US + CRP < 15	Sens. 92 (85–96), spec. 60 (54–65), PPV 47 (40–54), NPV 95 (91–97), LR+ 2.26 (1.95–2.60), LR− 0.14 (0.08–0.26)		

Negative US + PAS ≤ 5 + CRP < 5	Sens. 98 (92–99), spec. 41 (36–48), PPV 39 (34–46), NPV 98 (92–99), LR+ 1.66 (1.45–2.01), LR− 0.05 (0.03–0.15)		

US: ultrasound; CRP: C-reactive protein; PAS: pediatric appendicitis score; PPV: positive predictive value; NPV: negative predictive value; CI: confidence interval; LR: likelihood ratio; patients lacking PAS (*N* = 36) or CRP (*N* = 15) were not included.

## Abbreviations

CRP: C-reactive protein
LR: Likelihood-ratio
NPV: Negative predictive value
PAS: Pediatric appendicitis score
PPV: Positive predictive value
WBC: White blood cell.
